# Dogs (*Canis lupus familiaris*) are susceptible to the Kanizsa’s triangle illusion

**DOI:** 10.1007/s10071-021-01533-0

**Published:** 2021-07-16

**Authors:** Miina Lõoke, Lieta Marinelli, Cécile Guérineau, Christian Agrillo, Paolo Mongillo

**Affiliations:** 1 Laboratory of Applied Ethology, Department of Comparative Biomedicine and Food Science, University of Padua, Piazzetta del Donatore, 4, 35020 Legnaro, Italy; 2 Department of General Psychology, University of Padua, 35131 Padua, Italy; 3 Padua Neuroscience Center, University of Padua, 35131 Padua, Italy

**Keywords:** Dog, Illusory contours, Perception, Vision, Visual illusions

## Abstract

The ability to complete partially missing contours is widespread across the animal kingdom, but whether this extends to dogs is still unknown. To address this gap in knowledge, we assessed dogs’ susceptibility to one of the most common contour illusions, the Kanizsa’s triangle. Six dogs were trained to discriminate a triangle from other geometrical figures using a two-alternative conditioned discrimination task. Once the learning criterion was reached, dogs were presented with the Kanizsa’s triangle and a control stimulus, where inducers were rotated around their centre, so as to disrupt what would be perceived as a triangle by a human observer. As a group, dogs chose the illusory triangle significantly more often than control stimuli. At the individual level, susceptibility to the illusion was shown by five out of six dogs. This is the first study where dogs as a group show susceptibility to a visual illusion in the same manner as humans. Moreover, the analyses revealed a negative effect of age on susceptibility, an effect that was also found in humans. Altogether, this suggests that the underling perceptual mechanisms are similar between dogs and humans, and in sharp contrast with other categories of visual illusions to which the susceptibility of dogs has been previously assessed.

## Introduction

Visual perception is the result of the brain’s processing of sensory signals originating in retinal photoreceptors (Feng et al. 2017). Perception does not always correspond with the physical reality and stimuli that systematically trigger a misperception are commonly known as “visual illusions” (Gregory 1968). Discussion of visual illusions dates back to Aristotle and nowadays a variety of illusions tackling different perceptual mechanisms have been scientifically investigated (Eagleman 2001). For instance, recent literature provides experimental evidence about visual illusions eliciting misperception of relative size (Agrillo et al. 2019; Doherty et al. 2010), quantity (Agrillo et al. 2016; Kirjakovski and Matsumoto 2016), colour (Rizzi and Bonanomi 2012; Schlaffke et al. 2015), brightness (Blakeslee and McCourt 2012) and motion (Ashida et al. 2012; Kanazawa et al. 2013). Most of the studies in this field have concentrated on humans, but it has been suggested that some of the underlying mechanisms of illusory perception might be shared across species (Feng et al. 2017). Exploring the susceptibility to illusions by non-human animals may shed light on the top–down cognitive mechanisms of visual perception as well as providing new accounts about the phylogenesis of visual processing.

One of the most well-known classes of visual illusions is illusory contour. Contour illusions emerge when the visual scene fails to give complete luminance, texture, and/or colour cues on the boundaries of the objects, but the latter are nevertheless perceived (Kanizsa 1976). Since the observer perceives visual elements that are physically absent, the contour illusions can be classified as “fiction” illusions (Gregory 1997). Common examples of fiction illusions are the Ehrenstein illusion and the Kanizsa’s triangle illusion. The first one is composed of radial line segments with abrupt ends, which results in the perception of a white disk covering the centre of the image, from where the rays virtually originate (Ehrenstein 1987). The latter consists of an arrangement of three circles with a missing sector (i.e. “pac-men” figures), with their centres placed at the corners of an imaginary triangle, and symmetrically pointing inwards with their open 60° angles. Typically, human observers perceive a white triangle in between such figures, by mentally filling in the missing parts of the edges (Kanizsa 1976). Besides human observers, various non-human species were shown to be susceptible to the Kanizsa’s illusion, including chimpanzees (Fagot and Tomonaga 2001), macaque monkeys (Feltner and Kiorpes 2010; Zimmermann 1962), cats (Bravo et al. 1988), mice (Okuyama-Uchimura and Komai 2016), fish (Fuss et al. 2014; Sovrano and Bisazza 2009; Wyzisk and Neumeyer 2007) and even insects (Horridge et al. 1992; Sakiyama and Gunji 2016). Most of these studies have used two-alternative forced-choice tasks, in which animals were trained to discriminate figures with whole contours, after which their ability to transfer the discrimination to figures with seemingly occluded contours was assessed in transfer tests. Slight differences may exist between experiments in the discriminants or in characteristics of the inducers. For instance, Okuyama-Uchimura and Komai (2016) trained mice to discriminate rectangles based on their orientation, whereas Sovrano and Bisazza (2009) trained fish to discriminate a triangle and a square on different backgrounds. The perception of contour illusions is rendered possible due to the ability of perceptual completion (Nanay 2018; Nieder 2002). Besides contour illusions, perceptual completion also allows an observer to perceive objects with partially occluded or only partly illuminated contours, which are common in natural scenes (Nieder 2002). Therefore, possessing the ability of perceptual completion allows an animal to inspect its surrounding faster, which gives an animal a considerable advantage, particularly in hunting and in avoidance of predators. Altogether, this suggests that the ability to perceive illusory contours might be an ecologically important trait, which evolved early in evolution and was conserved across phylogenetically distant species. Indeed, neurophysiological recordings in primates have revealed that the illusory figures evoke cortical activation amongst the phylogenetically oldest parts of central visual pathways, namely the primary visual cortex (V1) and the secondary visual area (V2) (Grosof et al. 1993; von der Heydt et al. 1984; Lee and Nguyen 2001; Ramsden et al. 2001).

Studies on the perception of the Kanizsa’s triangle in humans have shown that the strength of the perception is positively correlated with the support ratio, i.e. the ratio between the physically specified contours and the total edge length (Halko et al. 2008; Otsuka et al. 2004; Shipley and Kellman 1992). When the support ratio is very low, the illusory contours are no longer perceived (Shipley and Kellman 1992). The principle is not limited to the Kanizsa’s triangle illusion. For instance, Petry and colleagues (1983) reported that the perceived brightness and sharpness of the white disk in the Ehrenstein illusion are affected both by the number and the thickness of the rays. When both of the variables are low (i.e., few thin lines are used as inducers), resulting in a very low support ratio, human observers report a weak perception of the inner disk. This phenomenon expands also to other species since rhesus monkeys presented a considerable decline in susceptibility of the Kanizsa’s illusion, when the support ratio was decreased to 25% (Feltner and Kiorpes 2010).

Dogs are increasingly used in cognitive studies due to their particular evolutionary history (Hare et al. 2002), comparative standpoint with humans (Miklósi et al. 2007) and applied value as working dogs. In particular, visual perception is a key feature in all of the above-mentioned aspects. However, our knowledge regarding dogs’ visual perception is far from being comprehensive. Several studies have recently explored dogs’ susceptibility to visual illusions, but most of them are confined to the so-called “distortion illusions” (Byosiere et al. 2017a,b, 2018; Keep et al. 2018; Lõoke et al. 2020; Miletto Petrazzini et al. 2017). Most of the studies have found that dogs do not perceive the illusions as humans do and the proposed explanation is the differences in global and local preference (Byosiere et al. 2020). In regard to fiction illusions, two studies have explored dogs’ susceptibility to contour illusions (Byosiere et al. 2017a, 2019). Byosiere and colleagues (Byosiere et al. 2017a) studied dogs´ susceptibility to an illusory contour version of the Ebbinghaus–Titchener illusion, where the spatial relationship between a circle and surrounding elements results in a misperception of the circle’s size. Three dogs out of five showed susceptibility to the illusion, suggesting that they were able to perceptually complete the missing contours. However, besides the relatively low proportion of dogs susceptible to the illusion, dogs were trained to discriminate illusory figures of different size before being tested for their susceptibility, which leaves the doubt that dogs might have learned to discriminate the circle size based on the inducers without perceptually completing the edges. The second study (Byosiere et al. 2019) presented dogs with a classical version of the Ehrenstein illusion. As a group, dogs failed to show susceptibility to the illusion, but the individual results of two dogs out of six suggested that these subjects were able to perceive missing contours. One possible reason for the lack of susceptibility might be a small support ratio. Altogether, the above-mentioned studies suggest that dogs are scarcely susceptible to the contour illusions. If this holds true, it would raise new questions in the evolution of visual perception, since the ability of perceptual completion is highly conserved across species and dogs would stand out as an exception.

The current study aims to assess dogs’ susceptibility to illusory contours in the Kanizsa’s triangle illusion. Dogs were initially trained to discriminate a triangle from other geometrical figures and later tested in their ability to transfer this discrimination, when presented with the Kanizsa’s triangle and a proper control figure. If dogs are able to perceptually complete the missing contours, they are expected to choose the illusory triangle more often than expected by chance. The illusion had a support ratio of 0.67, that is known to result in a strong perceptual effect in humans. In fact, the Kanizsa’s triangle allows an easily manipulable support ratio and may provide a more effective tool for assessing the ability to perceptually complete missing contours. Moreover, the susceptibility to this illusion has been assessed in a higher number of species than the Ehrenstein illusion and therefore, it makes assessing Kanizsa’s triangle illusion in dogs a more suitable choice for comparative purposes.

## Materials and methods

### Subjects

The sample consisted of six pet dogs (Table 1). The three mixed breed dogs were all mesocephalic and medium-sized (height at the withers 45 to 55 cm). The dogs’ average age was 4.3 ± 2.6 years. The dog owners were students or workers of the University of Padua and were recruited on a voluntary basis. The criterion for dogs’ selection was a good health condition, willingness to cooperate in the laboratory setting and high motivation for food.

**Table 1:** Subjects demographics at the time of testing

Subjects’ number	Age (y)	Sex	Breed
1	9.2	F	Golden retriever
2	3.2	M	Mixed breed
3	4.8	F	Whippet
4	1.3	F	Mixed breed
5	5.3	M	Mixed breed
6	2.0	F	Golden retriever

### Experimental setting

The experiment was conducted in a quiet room (4.7 × 5.8 m) with dim light. Two identical touch screen monitors (VG248QE, ASUSTeK Computer Inc., Taipei, Taiwan) measuring 53 × 30 cm, were used for stimuli presentation, with the refresh rate set to 100 Hz. Monitors were positioned side by side, with 25 cm in between and the vertical middle point of both monitors was set at the eye level of each subject. Both monitors were connected to a PC (Optiplex 960, Dell Inc., Round Rock, Texas, USA) which was operated through a Bluetooth keyboard (Logitech K400R, Logitech International S.A. Losanna, Switzerland). A coloured tape on the floor at 120 cm from the middle point between monitors marked the position at which the dog’s head was kept during the presentation of the stimuli (see below for details). A chair for the experimenter was placed at either side of such mark.

### Stimuli

#### Training stimuli

White geometrical shapes presented on black background were used as training stimuli. These included an equilateral triangle, which was used as a positive stimulus. Ten additional shapes were used as negative stimuli, including a square, a cross, a circle, an arch, a half-moon, a heart, a hexagon and letters “L”, “T” and “C”. One side of the triangle measured 10.5 cm and was composed of 555 pixels. The triangle had an area of 47.7 cm^2^ and all negative stimuli had approximately the same size. All stimuli were presented in the middle of the screen. The stimuli were created with OpenSesame (version 3.2.8 *Kafkaesque Koffka,* Mathôt et al. 2012).

#### Test stimuli

**Fig. 1:**
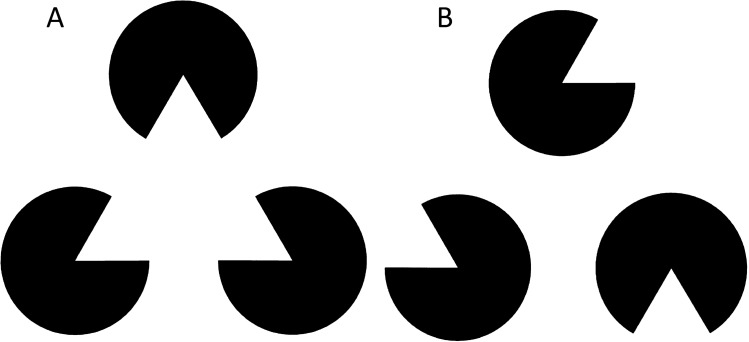
The illusory stimulus used in the test phase (**a**) and an example of the non-illusory stimulus used in the test phase (**b**)

All test stimuli consisted of three black circles with a missing sector of 60° (i.e. “pac-man” figures). The centre of the three pac-men was located in the same position as the apexes of the triangle of the training phase. The pac-men could be rotated so that the edges of the sectors of each pac-man were aligned with those of the other two pac-men, which typically generate the illusory perception of a solid triangle by humans (illusory stimulus; Fig. 1a). Alternatively, the pac-men were rotated, so that sectors were not aligned (non-illusory stimulus; Fig. 1b). Ten different non-illusory configurations were presented, including five figures where the three sectors had the same orientation as the illusory stimulus, but the pac-men were not in the same position as the illusory stimulus. All pac-men figures were presented on white background in the middle of the screen. The radius of the circle was 185 pixels, therefore the physical contours covered 2/3 (0.67) of the illusionary triangle sides.

### Experimental procedures

The experiment was composed of two phases, a training and a test phase. Dogs who were not accustomed to using the touch screen apparatus were previously involved in a preliminary procedural training. Two dogs (subjects 4 and 5) were already accustomed to using the touch screen apparatus. However, the stimuli were non-illusory and of a different nature (i.e. random dot motion displays); therefore, it is unlikely that the previous experience would have affected the results of the current experiment. The preliminary training was aimed at teaching dogs to wait at the designated initial position, visually inspect the monitors and touch the screen with their snout where a 4.5 cm plain white circle was presented on a black background. The preliminary training sessions were composed of 20 trials to accustom the dogs to the trial repetition. Preliminary training was completed when the dog was comfortable and accurately performing every aspect of the procedure. In the days in which dogs were involved in the study, they were only fed in the previous evenings to keep the dogs’ food motivation high.

### Training phase

The training phase used a two-alternative conditioned discrimination task to teach the dog to choose the triangle over other geometrical figures. The training phase consisted of sessions of 20 trials. Each trial started with the dog standing at the designated initial position beside the experimenter, who held it gently by its harness. Once the dog was oriented towards the monitors, the experimenter closed her eyes (Macpherson and Roberts 2013; Range et al. 2014) to avoid influencing the subjects’ choice and simultaneously started the presentation of the stimuli. The positive and the negative stimuli appeared on the monitors concurrently. The experimenter held the dog for 5 s to allow the dog to inspect the stimuli before choosing. Then, the experimenter said “Go!” and released the dog, who was then free to approach and touch one of the two monitors. Both stimuli disappeared upon touching one of the screens. If the dog chose the monitor with positive stimulus, it received a verbal praise and a food reward (either a commercial dog treat or a piece of sausage) tossed on the ground by the experimenter. After eating the food, the dog was called back to the starting position for the following trial. If the dog chose the negative stimulus, it was called back to the starting position without receiving any reward. If the dog did not make a choice within 60 s, the experimenter proceeded with the next presentation and the result was recorded as “No choice”.

The training phase was organized in steps with increasing difficulty to make learning easier for dogs. In the first step of the training phase, a black screen served as a negative stimulus. In the second step, a figure “L” was used as a negative stimulus. The third step had four negative stimuli presented in random order: the figure “L”, a square, a figure “T” and a cross. The fourth step had seven negative stimuli presented in a random order: a circle, a hexagon and an arch were added to the previous shapes. The final step had ten negative stimuli: a heart, a figure “C”, a half-moon and the previously mentioned shapes. Each time the dog made 2 or less mistakes (i.e., 90% accuracy) for 3 sessions in a row, it was moved to the next step. The side of the positive stimulus was counterbalanced across the session and semi-randomised so that it never appeared on the same side more than three times in a row. Each dog underwent a maximum of five training sessions per day, with an interval between sessions of at least 30 min. The dogs were moved to the test phase when they chose the positive stimulus for at least 18 out of 20 trials (i.e., 90% accuracy) in 6 consecutive sessions in the last step (i.e., using ten different negative stimuli), distributed over two separate days.

### Test phase

Test phase was meant to assess the dogs´ susceptibility to the Kanisza’s triangle illusion. Test phase sessions consisted of 25 trials, which included 20 training trials and 5 test trials. On test trials, both an illusory and non-illusory stimulus were presented. Test trials were regularly presented on every fifth trial and followed a similar procedure to the training trials, except that in the test trials, the dogs were reinforced randomly half of the times regardless of their choice. In the training trials of this phase, choices of the positive or negative stimulus had the same consequences as in the training phase. If the dogs made more than two mistakes in the training trials, they were moved back to the training phase, required to reach the learning criterion, and tested again. Each dog underwent five test sessions, therefore they were presented with 25 test trials overall. The time interval between sessions performed by the same dog was at least 30 min.

### Data collection and analyses

Data for the choice performed by the dogs in each trial, both in training and test phase, were automatically collected with OpenSesame. In addition, the data regarding the number of training sessions for each subject were collected. A two-tailed binomial test was run for each individual dog to test the null hypothesis (H0) that the choices were not different from chance level in the test trials. Only the test sessions where two or less mistakes were made in the training trials were considered in the final analysis. To assess whether an overall prevalence for choosing the illusionary stimuli was present in our sample, we performed a one-sample two-tailed Student’s *t* test on the dogs means for the type of choice (0 = non-illusionary stimulus, 1 = illusionary stimulus) expressed in the 25 test trials, testing the null hypothesis (H0) that the mean was equal to or lower than 0.5. To analyze if the length of the training phase or the age of the dog affected the choices in the test phase, a repeated measures binomial logistic regression analyses were computed. All statistical analyses were conducted using R (version 3.5.2; R Core Team 2018), with statistical significance level set at 0.05.

## Results

All six dogs reached the learning criterion in the training phase. The mean number of training sessions was 85 ± 26. The maximum number of training sessions was 129 and the minimum number was 60. Two dogs needed to go back to the training phase from the test phase. Both times the learning criterion was reached immediately within the next six sessions and the dogs were moved back to the test phase.

The individual results are presented in Table 2. All dogs made a choice in the test trials within 60 s and “No choice” was never recorded. The analyses revealed that five dogs chose the illusionary stimuli over chance level and one dog performed at chance level in the test phase. At the group level, dogs chose the illusionary stimuli more often than expected by chance (*t*(5) = 7.7, *p* < 0.001, Cohen’s d = 3.2).

**Table 2:** The number of training sessions, choices of the illusionary stimulus on 25 test trials (i.e., the entire test phase) and the *p* value of the two-tailed binomial test

Subject number	Nr of training sessions	*N* of choices of the illusionary stimulus on 25 presentations	Binomial test *p* value
1	102	16	0.230
2	60	22	< 0.001
3	69	21	0.001
4	68	24	< 0.001
5	129	23	< 0.001
6	81	22	< 0.001

The logistic regression revealed that older dogs chose the illusory stimuli significantly less often in the test phase (*z* = − 3.22, *p* = 0.001, Cohen’s d = 0.81). The length of the training phase had no effect on the test phase choices (*z* = 1.28, *p* = 0.20, Cohen’s d = 0.03). Due to the low number of choices of the non-illusory stimulus across the test sessions, we could not analyse statistically if the dogs’ performance changed across the test phase. However, the distribution of choices across test sessions did not reveal any consistent pattern.

## Discussion

This study provides compelling evidence that dogs are susceptible to a “fiction” illusion, i.e., the Kanizsa’s triangle illusion. Similar results have been obtained in other species, ranging from insects to primates (Bravo et al. 1988; Fagot and Tomonaga 2001; Feltner and Kiorpes 2010; Fuss et al. 2014; Horridge et al. 1992; Okuyama-Uchimura and Komai 2016; Sakiyama and Gunji 2016; Sovrano and Bisazza 2009). Therefore, the current finding aligns with the idea that perceptual completion of missing contours is widespread in the animal kingdom and that the dogs are no exception to this. However, the result is in sharp contrast with the general lack of susceptibility to the illusions so far observed in dogs (Byosiere et al. 2020).

The current results seem to clash particularly with previous work by Byosiere and colleagues (2019), who assessed dogs’ susceptibility to the Ehrenstein illusion. Contrary to the present results, ability to perceptually complete the illusory figure was only found in a minority of dogs in the sample, resulting in the failure to support susceptibility to illusion in dogs as a group. Although both the Ehrenstein and the Kanisza’s triangle illusions rely on perceptual completion, the higher susceptibility to the latter could be due to key differences in the structure of the two illusions. A first, crucial difference is that Kanizsa’s triangle inducers are of the “edge type”, whereby the edges of the inducers are collinear to the edges of the illusory shape. Conversely, in the Ehrenstein illusion, inducers are “line-end type”, meaning that the inducing lines are perpendicular to the illusory contour (Lesher and Mingolla 1993). It has been proposed that in humans, the neural underpinnings of these two illusions are slightly different: in the former, the neural activity along the edge is used directly for completion, whereas in the latter, the completion is divided in several stages, with neurons both responding to the end of lines and the line orientation (Lesher and Mingolla 1993). Moreover, in humans, it is well known that the perceptual strength of the Kanizsa’s triangle illusion is monotonically increased with the increase of the support ratio (Shipley and Kellman 1992). The perceptual strength of the Ehrenstein illusion depends both on the width and the number of inducing lines, whereas the perceived brightness shows an inverted U-function in relation to the inducer width (Petry et al. 1983). Even though the previous evidence shows that the support ratios are not directly comparable, there still remains a doubt that the support ratio used by Byosiere and colleagues (2019) was not sufficient for dogs to perceive the Ehrenstein illusion. In humans, the optimal support ratio for the Ehreinstein illusion is around 40%, and with a support ratio less than 20% the illusion is perceived weakly (Petry et al. 1983). However, the performance of two dogs of the study of Byosiere and colleagues did suggest the dog’s ability to perceive the Ehrenstein illusion and interestingly, they were the only two dogs that were trained to choose the smaller figure with a support ratio of 18.5%. Accordingly, the rest of the dogs trained to choose the larger figure with the support ratio of 8.5% were not susceptible to illusion (Byosiere et al. 2019). This seems to indicate that with an increase in the support ratio, the susceptibility to Ehrenstein illusion in dogs might be comparable to the one reported in the current study.

To date, no studies have found that dogs perceive visual illusions in a manner similar to humans. Previous studies exploring the perception of Ponzo, Müller-Lyer illusion, Solitaire, Ebbinghaus-Titchener illusion, and Delboeuf illusions conclude that dogs as a group are not susceptible to them or perceive them in the opposite manner to humans (Byosiere et al. 2017a,b, 2018; Keep et al. 2018; Lõoke et al. 2020; Miletto Petrazzini et al. 2017). It should be noted, however, that in all such illusions, collectively classified as the “distortion” illusions (Gregory 1991), the visual context elicits a misperception of either length, number or size. The susceptibility to such illusions is closely related with quantity estimation abilities, whereas the susceptibility to contour illusions lies more heavily on object recognition. As they require different capabilities, the illusions trigger the activation of different cortical regions. In humans, the contour illusions are predominantly processed in the cortical areas confined to the visual cortex, mainly in V1 and V2 (Ffytche and Zeki 1996; Seghier and Vuilleumier 2006). Conversely, the perception of distortion illusions requires also an integration of higher cortical areas responsible for numerical skills and magnitude estimation (Axelrod et al. 2017; Carther-Krone et al. 2020; Qiu et al. 2008; Weidner and Fink 2007). To date, it is unknown where exactly the visual illusions are processed in the canine brain. However, the results of behavioral experiments suggest that the mechanisms underlying perceptual completion are more similar between dogs and humans than mechanisms in which quantitative information has to be extracted from visual inputs.

While most of the dogs in the current study proved to be individually susceptible to the Kanizsa’s illusion, one dog did not. In view of the small sample size, it is difficult to make any conclusive discussion on underlying factors that may have contributed to this result. However, the only dog choosing at chance level was the oldest of the sample and possibly considered senior. There lies the possibility that the dog had a vision deficit that might have affected the results. However, such a hypothesis is unlikely, as all dogs went through a veterinary check prior to enrolling to the study; moreover, susceptibility to the Kanizsa’s triangle illusion does not seem to be affected by visual deficit produced by retinal scotomas (De Stefani et al. 2011). Furthermore, the statistical analyses of the current study found a general significant effect of age, indicating a lower probability to choose the illusory triangle by older subjects. Clearly, our sample size is not sufficient to draw any conclusive consideration of the role of age; however, it is interesting to highlight a potential parallel with humans, as elderly people are also less susceptible to the contour illusions compared to young adults (Kurtz 2011). One of the proposed reasons for elderly humans’ lower susceptibility of the Kanizsa’s triangle illusion (Kurtz 2011) is linked to a tendency to prioritize the processing of local elements, rather than the global level of hierarchical stimuli (Lux et al. 2008). Similar to humans, dogs seem to prioritize the global perception of hierarchical stimuli, although not as strongly and with a higher individual variability than humans (Pitteri et al. 2014). While no data exist about the effect of ageing on hierarchical stimulus perception in dogs, the same mechanism might be true for dogs.

Our sample consisted of dogs of various breeds. While the sample size is certainly too small to draw conclusion about the generalizability of the result to the dog population at large, the results suggest that perceptual completion may be shared across breeds. However, while we did include both mesocephalic and dolichocephalic dogs, none of our tested subjects was brachycephalic. It should be noted that the study by Byosiere and collaborators (2019) on the Ehrenstein illusion involved only Lagotto Romagnolo dogs, a moderately brachycephalic breed. In view of the demonstrated influence of cranial morphology on dogs’ use of visual information (McGreevy et al. 2004), the possibility that the sample composition in terms of breed may have contributed to the difference in susceptibility found in the two studies must be taken into account.

## Conclusion

Despite several differences in illusory perception, the finding of the present study indicates that dogs are susceptible to illusory contours. The contextual lack of difference to other illusions by dogs, reinforces the idea that the cortical mechanisms underlying illusory contours are different from that related to “distortion” illusions. Our results also demonstrate that, despite several differences between humans and dogs in perceptual mechanisms, at least the ones concerning illusory contours are similar. One potential aspect of similarity between dogs and humans which was only suggested by our results is a detrimental effect of ageing on the susceptibility to this illusion. Further experiments will be needed to determine if this is confirmed. Moreover, future research should explore the extent to which susceptibility to illusory contours can be extended to dogs with peculiar characteristics with known influence on visual processing, such as brachycephalism.
